# Endometrial Cancer Type 2 Incidence and Survival Disparities Within Subsets of the US Black Population

**DOI:** 10.3389/fonc.2021.699577

**Published:** 2021-07-20

**Authors:** Paulo S. Pinheiro, Heidy N. Medina, Tulay Koru-Sengul, Baozhen Qiao, Maria Schymura, Erin N. Kobetz, Matthew P. Schlumbrecht

**Affiliations:** ^1^ Sylvester Comprehensive Cancer Center, University of Miami School of Medicine, Miami, FL, United States; ^2^ Department of Public Health Sciences, University of Miami School of Medicine, Miami, FL, United States; ^3^ New York State Cancer Registry, New York State Department of Health, Albany, NY, United States; ^4^ Department of Medicine, University of Miami School of Medicine, Miami, FL, United States; ^5^ Department of Obstetrics & Gynecology, University of Miami School of Medicine, Miami, FL, United States

**Keywords:** endometrial cancer, uterine cancer, Blacks, incidence, survival, Black Hispanic, Caribbean, US-born

## Abstract

**Introduction:**

Endometrial cancer type 2 (EC2) carries a worse prognosis compared to EC type 1. EC2 disproportionately affects Black women among whom incidence is higher and survival is poorer compared to Whites. Here we assessed EC2 incidence and survival patterns among US Black ethnic groups: US-born Blacks (UBB), Caribbean-born Blacks (CBB), and Black Hispanics (BH).

**Methods:**

We analyzed population-based data (n=24,387) for the entire states of Florida and New York (2005–2016). Hysterectomy-corrected EC2 incidence rates were computed by racial-ethnic group, and survival disparities were examined using Cox regression adjusting for tumor characteristics, poverty level, and insurance status.

**Results:**

EC2 incidence rates were highest among UBB (24.4 per 100,000), followed by CBB (18.2), Whites (11.1), and Hispanics of all races (10.1). Compared to Whites, the age-adjusted cause-specific survival was worse for non-Hispanic Blacks (aHR: 1.61; 95%CI 1.52–1.71) and Hispanics of all races (aHR:1.09; 95% CI:1.01–1.18). In relation to Whites, survival was worse for non-Hispanic Blacks: UBB (aHR:1.62; 95%CI 1.52–1.74) and CBB (aHR:1.59; 95% CI:1.44–1.76) than for BH (aHR:1.30; 95% CI:1.05–1.61). Surgical resection was associated with a lower risk of death, while carcinosarcoma subtype and advanced stage at diagnosis were associated with a greater risk.

**Conclusions:**

Although higher EC2 incidence and lower survival are observed among all African-descent groups, there are significant intra-racial differences among UBB, CBB, and BH. This heterogeneity in EC2 patterns among Black populations suggests an interplay between genetic and socioenvironmental factors.

## Introduction

Uterine cancer incidence and mortality rates are increasing among US women (1, 2). Since 2016, cancer of the uterine body has been responsible for more than 10,000 deaths per year (3). Uterine cancer is diagnosed as endometrial cancer in 94% of cases, while 6% constitute uterine sarcomas (4). Endometrial cancer is quite heterogeneous and divided into the more often obesity-related -type 1 (EC1, representing low-grade endometrioid carcinomas) and EC type 2 (EC2, comprising carcinosarcoma, serous, clear-cell, mixed-cell, and high-grade endometrioid carcinomas) (5–7). -In contrast to EC1, which is associated with high 5-year survival, EC2 presents at a more advanced stage at diagnosis and is -associated with moderate to low survival after 5 years (7, 8).

Among upward-trending cancers, increasing incidence rates tend to exceed mortality rate increases because of better treatment modalities and earlier detection trends over time. This is not the case for uterine cancer, for which mortality is increasing faster than incidence (1, 2). This is a result of examining all subtypes of endometrial cancer as one homogenous group. EC1 is the more common type accounting for 60% of all new endometrial cancer cases, but it only accounts for a small proportion of endometrial-specific deaths (25%) (7). On the contrary, the less common EC2 accounts for nearly 75% of all endometrial cancer deaths (7). As previously shown, the incidence of the less common EC2 is increasing faster than EC1 (8), resulting in a shift in the severity of endometrial cancer overall. Confirming this complex trend, survival for all endometrial cancers combined has not shown an improvement in the last decades (2, 9).

In addition to the EC1 and EC2 heterogeneity, the epidemiology of uterine cancer on a population basis is further complicated by the difficulty in assessing the true population at risk. The prevalence of hysterectomy, a procedure commonly performed to treat fibroids, menorrhagia, and endometriosis, is currently decreasing although it has historically been the second most common gynecological surgical procedure after cesarean section (10, 11). In the US, the prevalence of this procedure differs substantially by geography and race, with Black women having a higher prevalence of hysterectomy (6, 12–14).

Despite that, Black women in the US share a disproportionately higher burden and mortality rate for endometrial cancer (1, 14), including a higher proportion of EC2 cases and worse survival for all EC2 subtypes in comparison to other races (7, 8).

EC2 is not a common cancer, and little is known in regard to the specific genetic and environmental factors that may impact its risk (incidence) and prognosis (survival). On a population basis, studies on incidence and survival of EC2 have been few, and none have scrutinized the intra-racial diversity in patterns among the most afflicted group in the US, women of African descent, particularly non-Hispanic Blacks. To our knowledge, only two publications have reported population-based rates of endometrial cancer among US Black populations (15, 16) including US-born, Caribbean-born, and African-born populations. One study has shown similar mortality rates for endometrial cancer across all three populations, which is suggestive of similar vulnerability between these populations (15). However, no research has studied incidence or survival for EC2, specifically in populations of African descent.

In this study, we aim to examine the incidence and survival of the more aggressive EC2 in the three largest racial-ethnic groups in the US, that is, non-Hispanic Whites, non-Hispanic Blacks, and Hispanics, and to explore the intra-racial differences in populations of African descent, namely, US-born Blacks, Caribbean-born Blacks, and Black Hispanics. The main hypothesis under study is that incidence and survival rates are different among all US Black populations, as well as between them and White and Hispanic women.

## Methods

### Source of Data

Data for all EC2s diagnosed in the states of Florida and New York (2005–2016) with primary site codes C54.X and C55.9 and morphology codes 8000–8951 per the International Classification of Diseases for Oncology, third edition (ICD-O-3), were obtained from the respective state cancer registries. Cancer registries routinely record sociodemographic characteristics such as age, race-ethnicity, census tract poverty level, and insurance type; as well as tumor characteristics such as stage at diagnosis, morphology, and grade. Only the three main racial-ethnic groups were studied in the current study: non-Hispanic Whites, non-Hispanic Blacks, and Hispanics (of any race), from now on referred to as Whites, Blacks, and Hispanics, for simplicity. Consistent with previous studies, morphology was categorized according to previous studies as either EC1 (low-grade endometrioid) or EC2 (high-grade endometrioid, clear cell, mixed cell, carcinosarcoma, and serous) (5–8). For incidence calculations, unspecified high-grade adenocarcinomas (morphology code 8140) were proportionally allocated for each racial-ethnic and age-group into high-grade endometrioid, clear-cell, and serous carcinomas in Clarke et al. (14).

### Classification of Populations of Non-Hispanic African Descent

Florida and New York data were chosen because these two states combined include 65% of the Caribbean-born Black population in the US (2.1 million) (17). For intra-racial (intra-Black) categorization among non-Hispanic Blacks, cases were classified as US-born Blacks or Caribbean-born Blacks based on country of birth, following previous work on these populations (15, 16). Country of birth missingness is a problem in cancer incidence data (18). In our datasets, 75% of all EC2 cases among non-Hispanic Blacks had a known birthplace. However, in order to conduct accurate population-based comparisons, the inclusion of all (100%) Blacks is necessary, given that most cases with “unknown” birthplace are in fact US- or Caribbean-born, had their birthplace been recorded. To overcome this problem, we assigned those with a missing birthplace to the categories of US-born Blacks, Caribbean-born Blacks, and other non-Hispanic Blacks (born in Africa, Europe, etc.) according to the majority group in the 5-year age group and area of residence of each case with missing country of birth. Census tract of residence was used in the case of counties with more than 500,000 total population, and county of residence if the overall population in the respective county was less than 500,000.

### Statistical Analyses

For the incidence analysis, we calculated hysterectomy-uncorrected and corrected endometrial cancer rates for all EC2s combined, as well as corrected rates by morphology subtype and racial-ethnic group for the entire 2005–2016 period. Detailed population denominators for each race-ethnicity by state were obtained from the US Census Bureau, using pooled single-year American Community Survey (ACS) data for 2005–2016 (17). Hysterectomy data were retrieved from the Biannual Behavioral Risk Factor Surveillance System (BRFSS) survey for 2006–2016 (19). Due to its biennial feature, hysterectomy prevalence was assumed the same for the BRFSS survey year and the immediately preceding year. For example, BRFSS 2006 hysterectomy data was used for 2005–2006 and 2016 data for 2015–2016. Hysterectomy-corrected denominators were estimated separately by state, racial-ethnic group, and for each 2-year period and then pooled for both states. BRFSS hysterectomy proportions were obtained for Whites, Blacks, and Hispanics (of any race) for the states of Florida and New York. For non-Hispanic Blacks, we also pulled hysterectomy prevalence for two additional geographic levels: 1) Metro statistical areas (MSAs) of New York-Newark-Jersey City and Miami-Fort Lauderdale-Pompano Beach; these MSAs are the areas that comprise sizeable proportions of both Caribbean-born Blacks and US-born Blacks, while in the states of Florida and New York outside of these MSAs, US-born Blacks nearly exclusively account for the total Black populations; and 2) the South Atlantic Censal Region States that includes Florida and the Northeastern Censal Region States that includes New York. The statewide hysterectomy proportions by age-groups were used for Whites, Hispanics, and Blacks (all combined).

Since BRFSS only provides proportions of hysterectomies for all non-Hispanic Blacks, to distinguish between US- and Caribbean-born, we proceeded as follows. First, for each state, we assumed that the hysterectomy proportion for US-born Blacks in both the MSA and the remaining area of each state outside the MSA was similar to that of Blacks in the larger Censal Regions (a population with a very large weight of US-born Blacks and very low weight of Caribbean-born Black populations). Based on this assumption, while taking into account the age-specific population proportions of both US- and Caribbean-born Blacks in each MSA from the ACS (17), and the total hysterectomy prevalence among Blacks in each MSA, we estimated the age-specific hysterectomy proportions for Caribbean-born Blacks. Incidence rates for all EC2 combined by subtype and by racial-ethnic groups were calculated per 100,000 persons, annualized, and age-standardized to the 2000 US Standard Population using 18 age-group bands. Gamma intervals modification was used to calculate 95% confidence intervals. Finally, we used negative binomial regression with adjustment for age to compare the incidence rates by racial-ethnic group and according to the period of diagnosis (2005–2010 *versus* 2011–2016).

For survival analysis, only the first primaries of EC2 diagnosed during 2005–2016 in both states were included. For each of the Black women for whom a specific Black ethnicity (US-born and Caribbean-born Black) was unable to be determined, the assignment was allocated to the larger of the two populations in the county/census tract of residence. In computing survival times, we used the presumed alive assumption (18), whereby cases that were not found as deceased on successive annual mortality linkages were censored on the last date covered, in this case, December 31, 2016. Cause-specific survival time was thus measured in months from the date of diagnosis until the date of death from uterine cancer, or December 31, 2016, whichever occurred first. Cases with death by a cause other than uterine cancer according to the SEER definition for site-specific cause-of-death (20) were censored at the time of death. Patients diagnosed with morphologies 8140 (adenocarcinoma not otherwise specified), those of unknown grade, and those diagnosed at autopsy only or by death certificate were excluded. Cause-specific Cox-proportional regression models for overall survival with socio-demographics, tumor, and treatment-related variables were fit for race-ethnicity as the main effect in each model. For any combination of variables, four different models were considered where only the race-ethnicity variable was changed based on different classifications and subgroups of Blacks. Adjusted hazard ratio (aHR), corresponding 95% confidence interval (CI), and p-value were calculated. For each model, we tested the proportionality assumption both visually with Kaplan-Meier survival curves by race-ethnicity and also by fitting time-varying Cox models and testing the time-varying terms in the models. All models satisfied the assumptions of proportionality except a very minor deviation for the model that included the race-ethnicity variable with Black groups only as can be seen in the Kaplan-Meier curve.

Lastly, for Black Hispanics, a unique group within Hispanics which is rarely studied, incidence rates were not estimated due to the current gross under-recognition of Black race among Hispanics (18), in which case, incidence rates on a population basis would be impossibly low. Previous surveys have found that Hispanics tend not to report a race as often as identifying a common ethnicity, Hispanic/Latino, and only one in every four Afro-Latinos report being of Black race (21). Notwithstanding this, we opted to show the relevant characteristics for Black Hispanics in both states when the race was known and to study survival comparisons between Black Hispanics and non-Hispanic Blacks (US-born and Caribbean-born), with the underlying knowledge that data for this group are incomplete and possibly subject to some degree of bias. This study is in compliance with the Florida Department of Health (DOH) Institutional Review Board and has been approved by the New York State DOH. Data management and statistical analyses were conducted using SAS v9.4 software (SAS Institute Inc., Cary, NC, USA).

## Results

A total of 24,387 cases of EC2, which included 15,938 Whites, 5,260 Blacks, and 3,189 Hispanics, in FL and NY were analyzed. Among Black women, there were 3,568 US-born and 1,381 Caribbean-born Blacks. Only 353 Hispanics were recorded as being of Black racial background. High-grade endometrioid was the leading EC2 morphological type among Whites (35%) and Hispanics (32%), followed by serous subtype (23 and 28%, respectively). For both non-Hispanic Blacks and Black Hispanics, the predominant morphological type differed with serous carcinoma being the leading type for US-born Blacks (34%), Caribbean-born Blacks (36%), and Black Hispanics (32%) (*p*=0.346). Carcinosarcoma was the second most common morphological type for US-born and Caribbean-born Blacks, and a similar proportion was found among Black Hispanics (26, 26, and 25%, respectively) *(p*=0.939) (**Table 1**). Proportions of carcinosarcomas were lower among Whites and Hispanics (17 and 18% respectively) (*p*<0.01). Localized stage at diagnosis was more common among Whites (47% localized and 16% distant stage) (*p*<0.01), whereas distant stage was disproportionately recorded among Blacks (36% localized and 22% distant). Whites were more likely to have private insurance, while Hispanics and Caribbean-born Blacks had higher proportions of Medicaid beneficiaries and uninsured. Black Hispanics and US-born Blacks had the highest proportion of women living in areas of high poverty (53 and 49%, respectively) in comparison to only 13% of White women (*p*<0.01).

**Table 1:** Demographic and Clinical Characteristics of Endometrial Cancer Type 2 by Race-Ethnicity in Florida and New York (2005–2016).

	WHITES	HISPANICS		BLACKS		
	Total n	All Hispanics (any race)^a^ n	Black Hispanics^b^ n	All Blacks^a^ n	US-born n	Caribbean-born^c^ n
**Total**	15,938	3,189	353	5,260	3,568	1,381
**Average Annual Population**	22,283,445	7,784,296	345,330	5,996,582	4,571,526	1,121,738
**% National Coverage of FL+NY**	11.3%	15.3%	53.7%	14.9%	12.4%	65.1%
**Median Age (years)**	68	66	66	67	67	67
**Age Range**	23-105	24-100	33-96	23-100	23-100	32-100
**Histology (p<0.001)^d^**						
High-Grade Endometrioid	5,562 (34.9%)	1,025 (32.1%)	91 (25.8%)	1,163 (22.1%)	826 (23.2%)	279 (20.2%)
Clear Cell	827 (5.2%)	193 (6.1%)	17 (4.8%)	308 (5.9%)	212 (5.9%)	75 (5.4%)
Mixed Cell	3,222 (20.2%)	511 (16.0%)	45 (12.7%)	638 (12.1%)	415 (11.6%)	182 (13.2%)
Carcinosarcoma	2,648 (16.6%)	576 (18.1%)	87 (24.6%)	1,329 (25.3%)	909 (25.5%)	354 (25.6%)
Serous	3,679 (23.1%)	884 (27.7%)	113 (32.0%)	1,822 (34.6%)	1,206 (33.8%)	491 (35.6%)
**Stage (p<0.001)^d^**						
Localized	7,516 (47.2%)	1,372 (43.0%)	143 (40.5%)	1,904 (36.2%)	1,279 (35.8%)	489 (35.4%)
Regional	5,403 (33.9%)	1,144 (35.9%)	136 (38.5%)	1,959 (37.2%)	1,321 (37.0%)	531 (38.5%)
Distant	2,463(15.5%)	540 (16.9%)	59 (16.7%)	1,169 (22.2%)	800 (22.4%)	307 (22.2%)
Unknown	556 (3.5%)	133 (4.2%)	15 (4.2%)	228 (4.3%)	168 (4.7%)	54 (3.9%)
**Insurance (p<0.001)^d^**						
Private	7,434 (46.6%)	1,054 (33.1%)	104 (29.5%)	1,824 (34.7%)	1,264 (35.4%)	449 (32.5%)
Medicare	6,239 (39.1%)	951 (29.8%)	111 (31.4%)	1,762 (33.5%)	1,238 (34.7%)	433 (31.4%)
Medicaid	1,051 (6.6%)	841 (26.4%)	116 (32.9%)	1,131 (21.5%)	718 (20.1%)	334 (24.2%)
No Insurance	297 (1.9%)	154 (4.8%)	8 (2.3%)	219 (4.2%)	134 (3.8%)	72 (5.2%)
Unknown	917 (5.8%)	189 (5.9%)	14 (4.0%)	324 (6.2%)	214 (6.0%)	93 (6.7%)
**Census Tract Poverty Level (p<0.001)^d^**						
Very Low	3,973 (24.9%)	355 (11.1%)	27 (7.6%)	478 (9.1%)	324 (9.1%)	133 (9.6%)
Low	4,830 (30.3%)	532 (16.7%)	37 (10.5%)	718 (13.7%)	483 (13.5%)	191 (13.8%)
Medium	4,897 (30.7%)	977 (30.6%)	103 (29.2%)	1,539 (29.3%)	993 (27.8%)	458 (33.2%)
High	2,129 (13.4%)	1,314 (41.2%)	186 (52.7%)	2,494 (47.4%)	1,741 (48.8%)	596 (43.2%)
Unknown	109 (0.7%)	11 (0.3%)	0 (0%)	31 (0.6%)	27 (0.8%)	3 (0.2%)
**State (p<0.001)^d^**						
FL	6,906 (43.3%)	1,693 (53.1%)	113 (32.0%)	2,026 (38.5%)	1,483 (41.6%)	523 (37.9%)
NY	9,032 (56.7%)	1,496 (46.9%)	240 (68.0%)	3,234 (61.5%)	2,085 (58.4%)	858 (62.1%)
**Treatment (p<0.001)^d^**						
Chemotherapy	6,152 (38.6%)	1,295 (40.6%)	183 (51.8%)	2,520 (47.9%)	1,641 (46.0%)	689 (49.9%)
Surgery	14,488 (90.9%)	2,880 (90.3%)	309 (87.5%)	4,503 (85.6%)	3,036 (85.1%)	1,186 (85.9%)
Radiotherapy	6,072 (38.1%)	1,180 (37.0%)	129 (36.5%)	1,862 (35.4%)	1,245 (34.9%)	493 (35.7%)

^a^Includes all cases of this race-ethnicity, not just listed groups; ^b^Top countries of birth: Cuba, Puerto Rico, Dominican Republic; ^c^Top countries of birth: Haiti, Jamaica, Trinidad and Tobago; ^d^p-value for chi-square tests comparing known categories among Whites, Black Hispanics, US-born Blacks, and Caribbean-born Blacks.

Age-adjusted incidence rates (uncorrected and corrected for hysterectomy) for EC2, by racial-ethnic group, are shown in **Table 2**, as well as corrected rates for EC2 subtypes. Based on the hysterectomy-corrected rates, EC2 was nearly twice as common among US-born Blacks compared to Whites (Incidence Rate Ratio (IRR) 1.93 95%CI 1.70–2.20) when adjusted for period of diagnosis and age (**Table 2**). Caribbean-born Black rates were lower than for US-born Blacks but significantly higher than for Whites (IRR 1.34 95%CI 1.16–1.54). By subtype, US-born Blacks had the highest rates for all EC2 subtypes, but especially for serous carcinoma and carcinosarcoma, more than three times the rates of White women. Caribbean-born Blacks also had double the rates of these two subtypes as well as higher rates of clear cell compared to Whites. Rates of mixed-cell and high-grade endometrioid carcinomas were not elevated in relation to Whites. Overall, comparing the older (2005–2010) and more recent period (2011–2016), hysterectomy-corrected rates show a significant increase in serous and mixed-cell carcinomas in all populations combined and a decrease in high-grade endometrioid and clear-cell carcinoma (**Table 2**). Baseline rates by race-ethnicity for each period can be seen in **Supplementary Table 1**.

**Table 2:** Age-adjusted^a^ total and hysterectomy-corrected^b^ incidence rates per 100,000 and rate ratios^c^ with 95% confidence intervals by race-ethnicity in Florida and New York (2005-2016).

	Total (Uncorrected)	Hysterectomy-Corrected					
	EC Type 2 total	EC Type 2 total	High-Grade Endometrioid	Serous	Carcinosarcoma	Mixed Cell	Clear Cell
	**Rates**						
WHITES	7.3 (7.2–7.5)	11.1 (10.9–11.2)	3.8 (3.7–3.9)	2.6 (2.5–2.6)	1.8 (1.7–1.9)	2.3 (2.2–2.4)	0.6 (0.5–0.6)
HISPANICS^d^	7.2 (7.0–7.5)	10.1 (9.8–10.5)	3.1 (2.9–3.3)	2.9 (2.7–3.1)	1.9 (1.7–2.0)	1.6 (1.5–1.7)	0.6 (0.5–0.7)
BLACKS^d^	14.2 (13.8–14.6)	23.5 (22.9–24.2)	4.9 (4.7–5.2)	8.4 (8.1–8.8)	5.9 (5.6–6.2)	2.8 (2.5–3.0)	1.4 (1.3–1.6)
* US-born*	14.8 (14.3–15.2)	24.4 (23.6–25.2)	5.3 (5.0–5.7)	8.6 (8.1–9.1)	6.2 (5.8–6.6)	2.8 (2.5–3.0)	1.5 (1.3–1.7)
* Caribbean-born*	12.6 (12.0–13.3)	18.2 (17.2–19.2)	3.7 (3.3–4.2)	6.7 (6.1–7.3)	4.6 (4.1–5.1)	2.2 (1.9–2.6)	1.0 (0.8–1.3)
	**Rate Ratios**						
Period of Diagnosis: 2011–2016 *vs.* 2005–2010	–	1.07 (0.97–1.17)	0.86 (0.78–0.95)	1.27 (1.17–1.38)	0.98 (0.89–1.07)	1.35 (1.23–1.48)	0.89 (0.80–0.99)
WHITES	–	1	1	1	1	1	1
HISPANICS^d^	–	0.87 (0.77–0.98)	0.80 (0.78–0.95)	1.12 (1.01–1.25)	1.00 (0.88–1.13)	0.67 (0.60–0.76)	1.08 (0.92–1.27)
BLACKS^d^	–	1.83 (1.63–2.05)	1.22 (1.09–1.38)	3.12 (2.84–3.43)	3.07 (2.75–3.42)	1.17 (1.04–1.31)	2.50 (2.19–2.85)
* US-born*	–	1.93 (1.70–2.20)	1.34 (1.15–1.56)	3.38 (3.09–3.70)	3.41 (3.05–3.81)	1.25 (1.13–1.39)	2.69 (2.31–3.13)
* Caribbean-born*	–	1.34 (1.16–1.54)	0.83 (0.69–1.00)	2.34 (2.10–2.62)	2.29 (1.99–2.63)	0.93 (0.80–1.08)	1.61 (1.27–2.05)

^a^Age-adjusted to the 2000 U.S. Standard Population; ^b^corrected for BRFSS survey-weighted estimates of hysterectomy prevalence; ^c^negative binomial regression rate ratios adjusted for age and period of diagnosis; ^d^Includes all cases of this race-ethnicity; not just listed groups.

**Figure 1:**
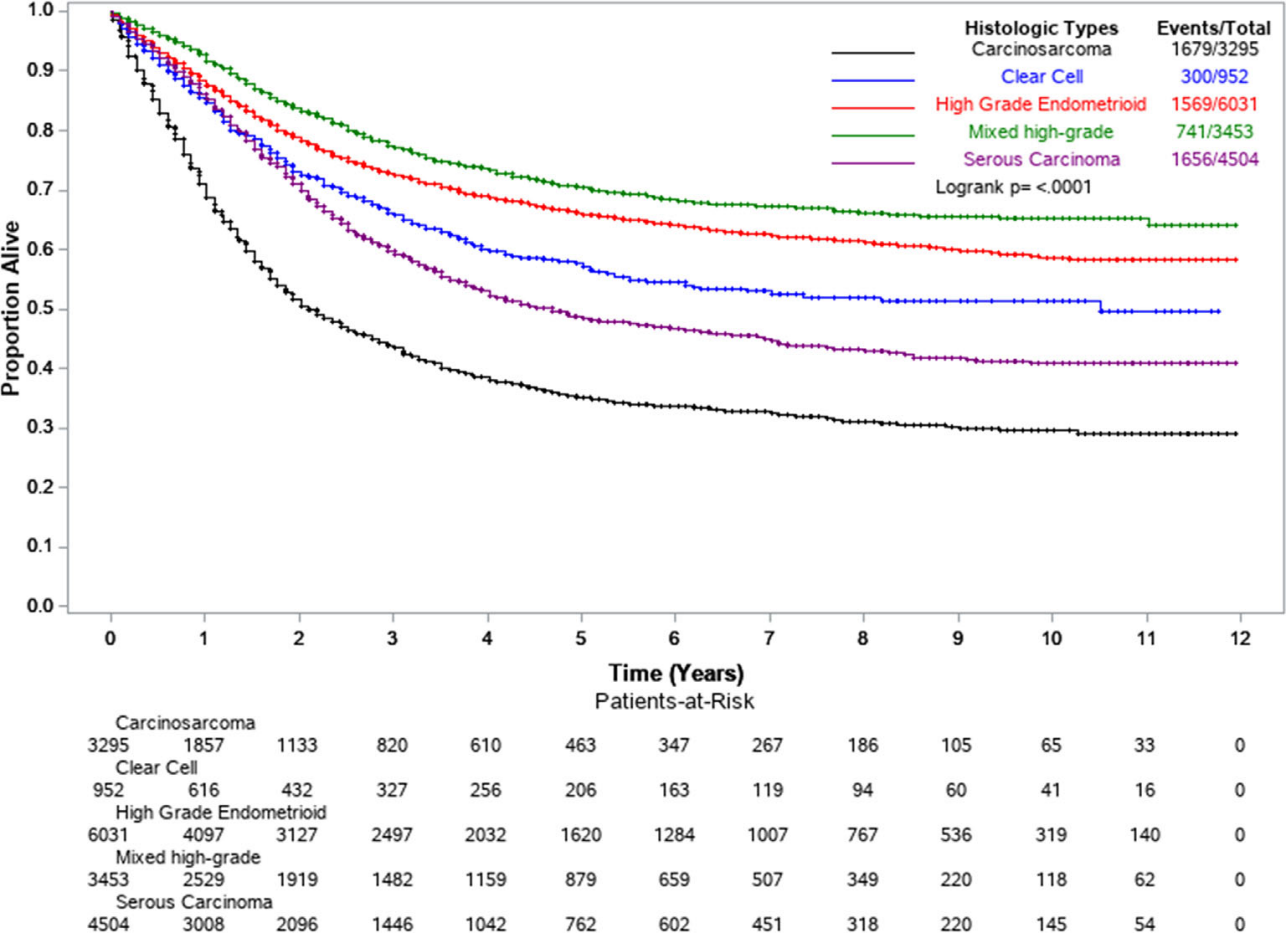
Kaplan-Meier survival curves by EC2 histological subtypes. Florida and New York, 2005–2016.

**Table 3** shows the results of Cox multivariable survival analysis performed on 18,246 EC2s first primary cancers among Whites, Blacks, and Hispanics. At the end of follow-up, 5,945 had died of uterine cancer, 11,369 were alive, and 932 had died of other causes and were thus censored at the date of death. Surgical treatment was recorded for 90.9% of all cases, while 45.7% received chemotherapy, 40.9% radiotherapy, and only 1.0% had any record of hormone therapy. In model 4, the full model adjusting for all variables, treatment and stage at diagnosis were the most important determinants of survival. Surgical resection (aHR=0.39, 95%CI: 0.36–0.42) was associated with a 61% lower risk of death over time; increased survival was also observed for those who were treated with radiation therapy (aHR=0.76, 95%CI: 0.72–0.81) and chemotherapy (aHR=0.79, 95%CI 0.75–0.84). Additionally, distant stage at diagnosis resulted in a much higher risk of death (aHR=7.63, 95%CI: 7.02–8.29) compared to those women diagnosed at localized stage. By EC2 subtype (**Figure 1** and **Table 3**, Model 4), carcinosarcoma was associated with a two-fold higher risk of death (aHR=2.01, 95%CI: 1.87–2.16) compared to the reference high-grade endometrioid, while serous carcinoma was associated with a 15% higher risk of death (aHR=1.15, 95%CI: 1.06–1.23).

**Table 3:** Hazard ratios (HR adjusted for state of residence) for demographic, social, and clinical determinants of Endometrial Cancer Type 2 survival in Florida and New York (2005–2016).

Prognostic Factors	Model 1	Model 2	Model 3	Model 4
	HR (95%CI)	HR (95%CI)	HR (95%CI)	HR (95%CI)
**Age**				
15–44	1 (Reference)	1 (Reference)	1 (Reference)	1 (Reference)
45–54	0.97 (0.78–1.20)	0.96 (0.77–1.20)	1.03 (0.83–1.28)	1.08 (0.87–1.35)
55–64	1.43 (1.17–1.75)	1.27 (1.04–1.55)	1.48 (1.21–1.81)	1.47 (1.20–1.79)
65–74	1.35 (1.11–1.65)	1.20 (0.98–1.47)	1.44 (1.18–1.76)	1.44 (1.17–1.76)
75+	2.16 (1.76–2.64)	1.87 (1.53–2.29)	2.24 (1.83–2.75)	1.95 (1.59–2.40)
**Histology**				
High Grade Endometrioid	–	1 (Reference)	1 (Reference)	1 (Reference)
Clear Cell	–	1.21 (1.07–1.37)	1.07 (0.94–1.21)	1.04 (0.92–1.18)
Mixed high-grade	–	0.85 (0.78–0.93)	0.86 (0.79–0.94)	0.87 (0.80–0.95)
Carcinosarcoma	–	2.42 (2.26–2.60)	1.94 (1.80–2.08)	2.01 (1.87–2.16)
Serous	–	1.48 (1.37–1.58)	1.12 (1.05–1.21)	1.15 (1.06–1.23)
**SEER Stage**				
Localized	–	–	1 (Reference)	1 (Reference)
Regional	–	–	2.86 (2.67–3.07)	3.13 (2.91–3.36)
Distant	–	–	8.44 (7.85–9.09)	7.63 (7.02–8.29)
Unknown	–	–	3.38 (2.91–3.92)	1.66 (1.41–1.95)
**Insurance**				
Private Insurance	–	–	–	1 (Reference)
Medicare	–	–	–	1.15 (1.08–1.22)
Medicaid	–	–	–	1.07 (0.99–1.16)
No insurance	–	–	–	0.95 (0.82–1.11)
Unknown	–	–	–	1.07 (0.95–1.19)
**Poverty Level**				
Very Low	–	–	–	1 (Reference)
Low	–	–	–	1.03 (0.95–1.11)
Medium	–	–	–	1.01 (0.94–1.09)
High	–	–	–	1.08 (1.00–1.18)
Unknown	–	–	–	0.76 (0.53–1.09)
**Chemotherapy**				
No	–	–	–	1 (Reference)
Yes	–	–	–	0.79 (0.75–0.84)
Unknown	–	–	–	0.86 (0.72–1.02)
**Surgery**				
No	–	–	–	1 (Reference)
Yes	–	–	–	0.39 (0.36–0.42)
Unknown	–	–	–	0.78 (0.56–1.10)
**Radiotherapy**				
No	–	–	–	1 (Reference)
Yes	–	–	–	0.76 (0.72–0.81)
Unknown	–	–	–	0.73 (0.62–0.86)
**Race/Ethnicity 1**				
WHITES	1 (Reference)	1 (Reference)	1 (Reference)	1 (Reference)
BLACKS^a^	1.61 (1.52–1.71)	1.40 (1.32–1.49)	1.31 (1.23–1.39)	1.22 (1.14–1.30)
HISPANICS^a^	1.09 (1.01–1.18)	1.05 (0.97–1.14)	1.03 (0.95–1.12)	1.00 (0.92–1.08)
**Race/Ethnicity 2** ^b^				
WHITES	1 (Reference)	1 (Reference)	1 (Reference)	1 (Reference)
* US-born Blacks*	1.62 (1.52–1.74)	1.42 (1.33–1.52)	1.36 (1.27–1.46)	1.25 (1.16–1.35)
* Caribbean-born Blacks*	1.59 (1.44–1.76)	1.36 (1.24–1.51)	1.23 (1.11–1.36)	1.14 (1.03–1.27)
* Black Hispanic*	1.30 (1.05–1.61)	1.15 (0.93–1.43)	1.05 (0.85–1.31)	0.98 (0.78–1.21)
**Race/Ethnicity 3** ^b^				
* US-born Blacks*	1 (Reference)	1 (Reference)	1 (Reference)	1 (Reference)
* Caribbean-born Blacks*	0.98 (0.88–1.09)	0.96 (0.86–1.07)	0.91 (0.81–1.01)	0.92 (0.82–1.02)
* Black Hispanic*	0.79 (0.63–0.98)	0.79 (0.63–0.99)	0.75 (0.60–0.94)	0.76 (0.61–0.95)

^a^Includes all cases of this race-ethnicity; not just listed groups; ^b^Hazard ratios obtained from separate models with only the mentioned racial/ethnic groups.

**Figure 2:**
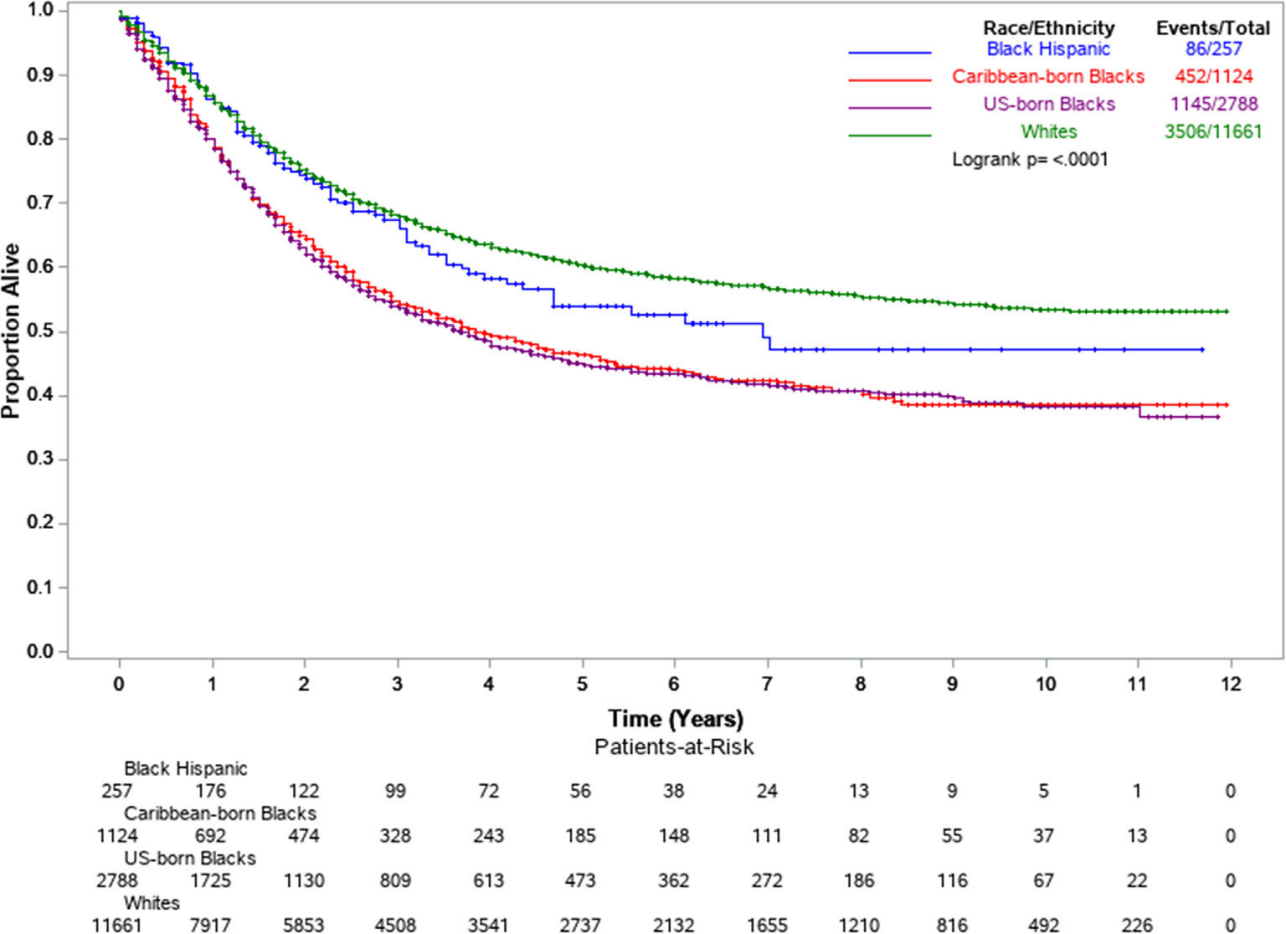
Kaplan-Meier survival curves by select racial-ethnic groups. Florida and New York, 2005–2016.

Cox regression models were extended to include intra-racial (US-born and Caribbean-born Blacks) and intra-ethnic groups (Black Hispanics). All Blacks combined showed an overall higher risk of death (model 1, aHR =1.61, 95%: 1.52–1.71) compared to Whites. However, this disadvantage was considerably reduced (model 2, aHR=1.22, 95%CI: 1.14–1.30) after adjustment for EC2 subtype, stage at diagnosis, and socio-economic and healthcare factors (poverty level, insurance, and treatment) in model 4. Among non-Hispanic Blacks, the results suggest some advantage for Caribbean-born Blacks with 8% lower endometrial cancer-specific survival (aHR=0.92, *p*=0.120) than US-born Blacks (**Table 3**, Model 4). Black Hispanics showed the lowest risk of death of all African-descent populations (**Figure 2**). After adjusting for all predictors, Black Hispanics had a 24% lower risk of death (model 4, aHR=0.76, 95% CI: 0.61–0.95) compared to US-born Blacks.

## Discussion

In this study, we analyzed population-based incidence and survival disparities specifically for EC2. This important subset of biologically heterogenous uterine cancers account for 75% of all deaths by endometrial cancer, require more aggressive treatment, and disproportionately affect Black populations. We found that the greater vulnerability of Blacks for EC2 reported previously extends to other women of African descent, regardless of region of origin and ethnicity. Black women not only have a higher incidence of EC2, especially of the more aggressive subtypes (carcinosarcoma and serous carcinoma), but also have a lower overall survival for EC2. While a race-wide vulnerability for EC2 is evident among all Black populations, there are intra-racial differences that suggest socio-environmental factors also play a role in the incidence and survival outcomes of EC2.

In terms of incidence, US-born Blacks showed a significantly higher rate of EC2 than Caribbean-born Blacks, followed by Whites and Hispanics. Median age at diagnosis was similar between US- and Caribbean-born Blacks; age-specific incidence rates for all age bands were either similar or lower for Caribbean-born Blacks (data not shown). However, there were important differences by subtype. Rates of serous, carcinosarcoma, and clear-cell carcinoma were higher among both US- and Caribbean-born Blacks in relation to Hispanics and Whites. Yet, while US-born Blacks still retained the highest incidence of high-grade endometrioid cancers and mixed-cell endometrial cancers, there was no significant difference between Whites and Caribbean-born Blacks for these subtypes. These results suggest two things: first that the increased “Black” vulnerability is for non-endometrioid EC2 subtypes; second, that US-born Blacks carry a higher risk for all EC2 subtypes. Thus, socio-environmental factors among US-born Blacks may partly determine their excess risk in relation to Caribbean-born Blacks who share a related racial background. Some of these factors may include experiences of racial discrimination and stress across the lifespan which start from a younger age for those who are US-born Blacks, varying levels of socioeconomic status, the built environment, and the respective impact these may have on diet, fertility, contraception, body mass index, and hormonal factors, which can be implicated in the risk and survival for EC2.

Our knowledge of the epidemiology and especially risk factors for these subtypes is limited. Little is known about the influence of genetic differences, obesity, diet, parity, and hormones on each EC2 subtype. Obesity has been more strongly linked to EC1 than EC2 (22, 23), while hormonal factors are not distinctively different between EC1 and EC2 (24). In any case, the factors determining the excess occurrence of carcinosarcoma and serous carcinoma especially among Black populations are unknown.

Other important findings include the overall increase in EC2 rates among Whites and Blacks over time and serous carcinoma among all groups (**Supplementary Table 1**), which can only be described as an unfavorable trend. The upside is that carcinosarcoma, the subtype associated with the worst prognosis of all EC2s, did not show an increase between 2005–2010 and 2011–2016, in contrast to previous reports (6, 8).

Survival disadvantages for EC2 were observed for all minority populations in relation to Whites according to age-only adjusted models (Model 1): 62% higher risk of death for US-born Blacks, 59% for Caribbean-born Blacks, 30% for Black Hispanics, and 9% for all Hispanics combined. However, after adjustment for morphology (Model 2), the differences between Whites and Hispanics were no longer significant, and the HRs for all Black populations were substantially reduced. This decrease was in line with the observed higher proportions of subtypes with worse prognosis, among Black populations: carcinosarcoma and serous carcinoma. The disadvantage observed among US- and Caribbean-born Blacks was further attenuated by potentially modifiable factors such as stage at diagnosis as well as more established modifiable ones (treatment, insurance, and poverty level), presented in the fully adjusted model (Model 4). While the difference is not significant, the fully adjusted model suggests some advantages for Caribbean-born populations in relation to US-born populations. Advantages for majority foreign-born populations such as Black Hispanics and Caribbean-born compared to US-born Blacks may result from the described healthy immigrant effect (25). For those of Hispanic ethnicity, highly jointed family structures may increase social support as described by the concept of “familismo” which has previously been suggested to have a role in cancer survival (26). Interestingly, when comparing all Hispanics combined (Race-Ethnicity 1) and Black Hispanics (Race-Ethnicity 2) with non-Hispanic Whites, as the common reference category, the HRs differ in models 1 and 2 but are similar in model 3. This suggests that the initial worse prognosis for Black Hispanics in comparison to Hispanics overall can be largely attributed to their differing prevalence of endometrial cancer subtypes and stage at diagnosis. Our results agree with the only existing survival study on endometrial cancer among individuals of African descent (27) that also suggested that Caribbean-born Blacks had a lower risk of death compared to US-born Blacks; however, this was a hospital-based study. In our study, the survival difference is much smaller (8% lower risk of death in this population-based analysis *versus* 35% in the hospital-based study). Influences of educational level, family connection, social support, and treatment compliance on endometrial cancer survival have not been analyzed in these heterogeneous Black populations, particularly among Black Hispanics, who show an advantage in relation to US- and Caribbean-born Blacks in this study.

The most notable strength of our study is its true population-based nature given that all cases of EC2 recorded in both states were included. Moreover, all rates were hysterectomy-corrected. Both registries have high-quality data according to NAACCR certifications (28). The depiction of intra-Black variability is novel, and to our knowledge, this is only the second time population-based incidence rates for Afro-Caribbeans in the US have been estimated (29). Moreover, we describe the experience of Black Hispanics, a unique group often ignored because of its smaller population size. By pooling data from Florida and New York, the representation of the two smaller Black populations, non-Hispanic Caribbean-born and Black Hispanics, is particularly robust, encompassing 65 and 53%, respectively, of all potential individuals of these racial-ethnic groups in the country. Lastly, we include all women of Black race regardless of missing birthplace, previously shown to be a variable not missing at random, avoiding a common selection bias shown to impact survival estimates (18) and underestimate incidence rates. Using the entire population, we also avoid the selection bias linked to healthcare access, which has been associated with hospital-based studies (30).

This study is not without limitations. The assignment into either the US- or Caribbean-born Black category for those with a missing birthplace could have been an important limitation in incidence and survival analyses. However, analyses pre- and post-group assignment showed nearly identical differences between the two groups. As an example, the incidence ratio between US-born and Caribbean-born Blacks for EC2 using total corrected rates only decreased slightly from 1.37 (pre-assignment, considering only 75% of all non-Hispanic Blacks) to 1.34 (considering 100%) as shown in **Table 2** (post-assignment). Similarly, for survival, in a direct comparison between US-born Blacks and Caribbean-born Blacks, the aHR did not differ substantially from 0.98 in model 1 shown in **Table 3** (post-assignment) to 0.97 (95%CI 0.85–1.08) (pre-assignment). The hysterectomy prevalence for Caribbean-born Blacks had to be estimated based on the metro areas of residence in contrast to areas where Blacks are mostly US-born. In terms of the survival analysis, the lack of clinical data on EC2 cases is a common limitation in cancer registry data. There is a lack of information on specific treatment modalities, such as specific surgical procedures performed, adherence, and completion of guideline-based care. Moreover, difficulties in follow-up can overestimate survival among foreign-born populations (18), which will somewhat underestimate the risk of death over time for Hispanics as a whole, including Blacks Hispanics, and Afro-Caribbeans. Additionally, other reported miscellaneous and unknown histologies of uterine cancer, which account for 3.0% for Whites, 4.3% for Hispanics, and 5.7% for Blacks, could correspond to EC2 and may underestimate an already high incidence for Black women. The increase in serous carcinoma among all populations could partly be due to better recognition of this subtype. Lastly, data on the molecular categorization of the various types of endometrial carcinoma and EC2 (POLE-ultramutated, microsatellite instability mutated, copy number high, and copy number low) (31) are not available in population-based cancer registries. However, studies evaluating associations between molecular signature and race-ethnicity mirror our findings, though further comprehensive study especially among these diverse populations of African descent is needed.

In conclusion, the need for research into EC2 subtypes, encompassing risk, and prognostic factors is a clear priority in the battle against this malignancy. Currently, from a clinical standpoint, therapeutic guidelines for the different subtypes of EC2 follow similar protocols despite the substantial differences in survival outcomes. The independent study and enrollment of these women with these cancers in clinical trials are made difficult since they are not common cancers. We found that all three Black populations analyzed had a higher risk of EC2 subtypes including serous, carcinosarcoma, and clear-cell carcinoma. Incidence and survival comparisons showed that US-born Blacks fared worse than other Black populations, thus emphasizing not only a genetic vulnerability common to all three populations but also socio-environmental factors that may constitute important modifiable factors in the battle against endometrial cancer. In this respect, research on epigenetic markers and related biological mechanisms, which may partly account for these differences, seems to be of particular interest for Black populations. There is a dearth of intra-racial and intra-ethnic health data for the US Black heterogeneous populations, which is surprising, given that this group bears a disproportionate burden of cancer morbidity and mortality (1, 2). Better knowledge of these intra-racial differences may allow us to find ways to better address endometrial cancer risk, early detection, and treatment challenges while enabling a better understanding of the epidemiology of this disease for all populations

## Data Availability Statement

The datasets presented in this article are not readily available because restrictions from Florida and New York Departments of Health apply to the availability of these data. The authors themselves are unauthorized to share the individual-level data. The datasets are available by request with required approvals from the respective state cancer registry programs and Departments of Health. Requests to access the datasets should be directed to Florida Department of Health Cancer Registry Program and Florida Department of Health Institutional Review Board. Applications for data request are available from the FCDS Webpage (http://fcds.med.miami.edu/inc/datarequest.shtml). Researchers should contact the New York State Cancer Registry at nyscr@health.ny.gov.

## Author Contributions

PP was involved in the conceptualization, methodology, formal analysis, writing of the original draft, review and editing of draft, and supervision of the study. HM was involved in the formal analysis, review and editing of draft, and visualization. TK-S was involved in the methodology, resources, and review and editing of draft. BQ and MS were involved in the resources, formal analysis, and review and editing of draft. EK was involved in the study supervision and review and editing of draft. MS was involved in the formal analysis, review and editing of draft, and study supervision. All authors contributed to the article and approved the submitted version.

## Funding

Supplemental funding was provided by the Sylvester Comprehensive Cancer Center at University of Miami and by the National Cancer Institute of the National Institutes of Health (NIH) under Award Number P30CA240139.

## Conflict of Interest

The authors declare that the research was conducted in the absence of any commercial or financial relationships that could be construed as a potential conflict of interest.
