# Supplementary material for: Endometrial Cancer Type 2 Incidence and Survival Disparities Within Subsets of the US Black Population
Source: Front Oncol. 2021 Jul 20;11:699577. doi: 10.3389/fonc.2021.699577 (PMC8329656; doi:10.3389/fonc.2021.699577)
Supplement: Supplementary file 1 [file Table_1.docx]

| **Supplementary Table 1. Age-adjusted^a^ hysterectomy-corrected^b^ incidence rates per 100,000 by race-ethnicity for Endometrial Cancer Type 2 subtypes in Florida and New York (2005-2016) and by period of diagnosis.** | | | | | | | |
| --- | --- | --- | --- | --- | --- | --- | --- |
|  | **Period of Diagnosis** | **EC Type 2 total** | **High-Grade Endometrioid** | **Serous** | **Carcinosarcoma** | **Mixed Cell** | **Clear Cell** |
|  |  | **Rate (95% CI)** | **Rate (95% CI)** | **Rate (95% CI)** | **Rate (95% CI)** | **Rate (95% CI)** | **Rate (95% CI)** |
| WHITES | 2005-2010 | 10.7 (10.4-10.9) | 4.2 (4.1-4.4) | 2.2 (2.1-2.3) | 1.8 (1.7-1.9) | 1.8 (1.7-1.9) | 0.6 (0.5-0.7) |
|  | 2011-2016 | 11.3 (11.0-11.5) * | 3.5 (3.4-3.7) ** | 2.8 (2.7-2.9) * | 1.8 (1.7-1.9) | 2.6 (2.5-2.7) * | 0.5 (0.5-0.6) |
| HISPANICS^c^ | 2005-2010 | 9.5 (9.0-10.1) | 3.3 (3.0-3.6) | 2.5 (2.2-2.8) | 1.9 (1.6-2.1) | 1.3 (1.1-1.5) | 0.6 (0.4-0.7) |
|  | 2011-2016 | 10.4 (9.9-10.9) | 3.1 (2.8-3.3) | 3.1 (2.8-3.4) * | 1.8 (1.6-2.0) | 1.8 (1.6-2.0) | 0.7 (0.6-0.8) |
| BLACKS^c^ | 2005-2010 | 22.3 (21.3-23.3) | 5.6 (5.2-6.1) | 6.9 (6.3-7.4) | 5.7 (5.2-6.2) | 2.5 (2.2-2.9) | 1.6 (1.4-1.9) |
|  | 2011-2016 | 24.2 (23.3-25.1) * | 4.6 (4.2-5.0) ** | 9.3 (8.7-9.9) * | 6.0 (5.6-6.5) | 3.0 (2.6-3.3) | 1.3 (1.1-1.6) |
| *US-born Blacks* | 2005-2010 | 24.1 (22.9-25.4) | 6.4 (5.8-7.1) | 7.1 (6.5-7.8) | 6.1 (5.5-6.8) | 2.7 (2.3-3.2) | 1.7 (1.4-2.1) |
|  | 2011-2016 | 25.6 (24.4-26.7) | 4.9 (4.5-5.5) ** | 9.8 (9.1-10.5) * | 6.5 (5.9-7.1) | 2.9 (2.5-3.3) | 1.4 (1.2-1.7) |
| *Caribbean-born Blacks* | 2005-2010 | 14.8 (13.5-16.2) | 3.4 (2.8-4.2) | 4.8 (4.1-5.6) | 4.0 (3.3-4.8) | 1.6 (1.2-2.2) | 1.0 (0.7-1.5) |
|  | 2011-2016 | 18.7 (17.4-20.2) * | 3.6 (3.0-4.3) | 7.0 (6.2-8.0) * | 4.5 (3.9-5.3) | 2.6 (2.1-3.2) | 1.0 (0.7-1.5) |
| a. Age-adjusted to the 2000 U.S. Standard Population; b. corrected for BRFSS survey-weighted estimates of hysterectomy prevalence; c. Includes all cases of this race-ethnicity; not just listed groups  *significant increase p<0.05  **significant decrease p< 0.05 | | | | | | | |
